# Obatoclax kills anaplastic thyroid cancer cells by inducing lysosome neutralization and necrosis

**DOI:** 10.18632/oncotarget.9121

**Published:** 2016-04-30

**Authors:** Devora Champa, Arturo Orlacchio, Bindi Patel, Michela Ranieri, Anton A Shemetov, Vladislav V Verkhusha, Ana Maria Cuervo, Antonio Di Cristofano

**Affiliations:** ^1^ Department of Developmental and Molecular Biology, Albert Einstein College of Medicine, Bronx, NY 10461, USA; ^2^ Department of Anatomy and Structural Biology, Albert Einstein College of Medicine, Bronx, NY 10461, USA

**Keywords:** thyroid cancer, lysosomes, necrosis, autophagy, obatoclax

## Abstract

Poorly differentiated and anaplastic thyroid carcinomas are very aggressive, almost invariably lethal neoplasms for which no effective treatment exists. These tumors are intrinsically resistant to cell death, even when their driver oncogenic signaling pathways are inhibited.

We have undertaken a detailed analysis, in mouse and human thyroid cancer cells, of the mechanism through which Obatoclax, a pan-inhibitor of the anti-apoptotic proteins of the BCL2 family, effectively reduces tumor growth *in vitro* and *in vivo*.

We demonstrate that Obatoclax does not induce apoptosis, but rather necrosis of thyroid cancer cells, and that non-transformed thyroid cells are significantly less affected by this compound. Surprisingly, we show that Obatoclax rapidly localizes to the lysosomes and induces loss of acidification, block of lysosomal fusion with autophagic vacuoles, and subsequent lysosomal permeabilization. Notably, prior lysosome neutralization using different V-ATPase inhibitors partially protects cancer cells from the toxic effects of Obatoclax. Although inhibition of autophagy does not affect Obatoclax-induced cell death, selective down-regulation of ATG7, but not of ATG5, partially impairs Obatoclax effects, suggesting the existence of autophagy-independent functions for ATG7. Strikingly, Obatoclax killing activity depends only on its accumulation in the lysosomes, and not on its interaction with BCL2 family members.

Finally, we show that also other lysosome-targeting compounds, Mefloquine and LLOMe, readily induce necrosis in thyroid cancer cells, and that Mefloquine significantly impairs tumor growth *in vivo*, highlighting a clear vulnerability of these aggressive, apoptosis-resistant tumors that can be therapeutically exploited.

## INTRODUCTION

Thyroid cancer is the most common endocrine malignancy and the fifth most prevalent cancer in women, with over 62,000 new cases estimated for 2015 [1]. The majority of thyroid tumors are successfully cured by surgical resection followed, in some cases, by radioactive iodine therapy [2]. Despite the overall benign prognosis, however, a subgroup of thyroid cancer histotypes, such as recurring well differentiated, poorly differentiated, and anaplastic tumors, behaves much more aggressively and often does not respond to chemotherapy, radiation therapy, or targeted therapy. Although these tumors have a relatively low frequency, they always carry a rather poor prognosis and thus represent a critical clinical issue.

A number of groups, including ours, have generated and characterized, over the past several years, an array of clinically relevant genetically engineered mouse strains and cells lines that faithfully recapitulate the genetic and clinical features of most types of human thyroid cancer [3–11]. These models represent unique and precious tools to address such critical issues as therapeutic target identification and validation [11–16], development and countering of therapy resistance [11, 17], as well as drawing a more precise blueprint of the signaling pathways commonly altered in thyroid cancer [4, 5, 9, 18–23].

We have recently reported that aggressive thyroid carcinomas are often characterized by the increased expression of anti-apoptotic proteins belonging to the BCL2 family, which might contribute to these tumors' resistance to antineoplastic agents [11]. In fact, we showed that Obatoclax, a small molecule designed as a pan-inhibitor of the BCL2 family members [24], was very effective in reducing thyroid tumor cell growth and inducing cell death both in cell culture and in an immunocompetent allograft model [11].

We have now characterized the mechanism of action of Obatoclax in thyroid cancer cells and present data supporting the surprising conclusion that its efficacy in our model systems is independent of BCL2 family member targeting. Instead, Obatoclax appears to localize to and functionally disrupt lysosomes, thus uncovering a critical vulnerability of thyroid cancer cells, which we show can be also targeted using additional, molecularly distinct, lysosome-disrupting small molecules.

## RESULTS

### Obatoclax induces necrotic cell death

**Figure 1:**
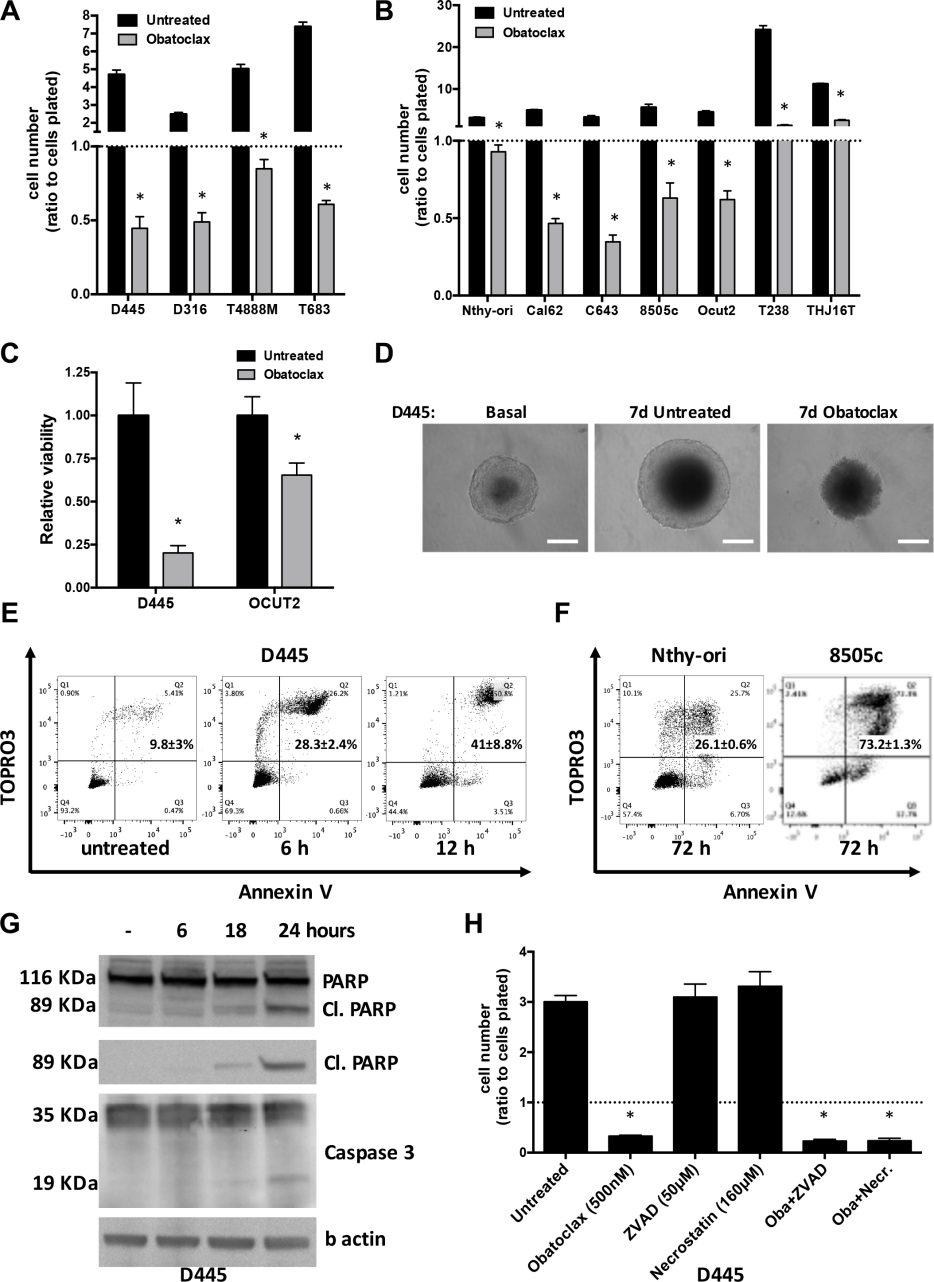
Obatoclax induces massive necrosis in mouse and human thyroid cancer cells Cell counts of (**A**) mouse thyroid cancer cell lines treated with Obatoclax (500 nM) for 24 hrs and (**B**) human thyroid cell lines treated with Obatoclax (1 μM) for 48 hrs. Counts were normalized to the number of cells at the time of treatment. (**C**) Effect of Obatoclax treatment on the viability of mouse and human thyroid cancer cells grown for 7 days as spheroids, determined by acid phosphatase assay. (**D**) Representative images of D445 cell spheroids treated with Obatoclax (1 μM) for 7 days. (E, F) Flow cytometric analysis of cell death in (**E**) D445 cells treated with Obatoclax (500 nM) for the specified times and (**F**) human thyroid cell lines treated with Obatoclax (1 μM) for 72 hrs. Numbers superimposed on the panels represent mean ± sd of three or more replicate experiments. (**G**) Western blot analysis to detect apoptotic markers in D445 cells treated with Obatoclax (500 nM) for the specified times. “Cl. PARP”: cleaved form of PARP. (**H**) Relative cell counts of D445 cells treated for 24 hrs with Obatoclax alone or after 1hr pretreatment with ZVAD or Necrostatin. Bars in graphs correspond to standard deviation.

To assess the ability of Obatoclax to kill thyroid cancer cells, we treated a panel of mouse and human cell lines, carrying different driver mutations (Table 1), with 500 nM and 1 μM Obatoclax, respectively. These concentrations were chosen based on the EC_90_ derived from preliminary WST1-based dose-response assays (data not shown). We then determined the ratio between the number of live cells at 24 or 48 hours post-treatment and the number of cells originally plated. While the number of untreated cells increased 2- to 25-fold, Obatoclax caused in almost all cell lines a striking reduction of the number of cells, well below plating levels, indicating strong cytotoxic activity (Figure 1A, 1B). In general, cells with RAS or BRAF activating mutations (D445, D316, T683, Cal62, C643, 8505c, Ocut-2) were the most sensitive, while SV40 T antigen-immortalized normal thyroid cells (Nthy-ori) and two of the three lines carrying *PIK3CA* mutations (T238, THJ16T) displayed only a cytostatic effect.

**Table 1:** Thyroid cancer cell lines used in this study

Cell Line	Species	Mutation(s)	Original tumor histology
D445	Mouse	*Kras*^G12D^ ; *Trp53*^−/−^	PDTC
D316	Mouse	*Kras*^G12D^ ;Trp53^−/−^	PDTC
T4888M	Mouse	*Pten*^−/−^; *Trp53*^−/−^	ATC
T683	Mouse	*Pten*−/−; *Kras*^G12D^	PDTC
Nthy-ori	Human	SV40 T antigen	normal
Cal62	Human	*KRAS*^G12R^; *Trp53*^mut^	ATC
C643	Human	*HRAS*^G13R^ ; *Trp53*^mut^	ATC
8505c	Human	*BRAF*^V600E^ ; *Trp53*^mut^	ATC
Ocut-2	Human	*BRAF*^V600E^ ; *PIK3CA*^H1074R^;	ATC
T238	Human	*BRAF*^V600E^ ; *PIK3C*^AE542K^; *Trp53*^mut^	ATC
THJ16T	Human	*PIK3CA*^E545K^ ; *Trp53*^mut^	ATC

Massive cell death was also observed when thyroid cancer cells were grown as tridimensional spheroids in ultralow attachment conditions. Seven days after exposure to Obatoclax, morphological analysis showed that spheroids were clearly smaller and darker than both basal and untreated ones, and the acid phosphatase assay revealed a significant reduction of cell viability (Figure 1C, 1D).

These data, which validate and further extend our previously published results, demonstrate that Obatoclax is highly effective in inducing cell death across a wide range of thyroid cancer cell lines.

Initial attempts to define the mode of death by staining cells with Annexin V-FITC and propidium iodide (PI) were unsuccessful, since Obatoclax-treated cells exhibited extremely distorted flow cytometric profiles (Supplementary Figure S1). This issue was explained by the finding that Obatoclax has strong autofluorescence, with very wide excitation and emission profiles that encompass both FITC and PI excitation/emission channels (see below). Thus, we elected to use TOPRO3 as a nuclear exclusion dye and Annexin V conjugated to Alexa Fluor 350 to avoid interference with Obatoclax autofluorescence.

When we analyzed Obatoclax-treated mouse thyroid cancer cells, we found that this compound induces massive necrosis (TOPRO3 and Annexin V double positive cells) as early as 6 hours after treatment, peaking between 24 and 48 hours, with no apoptotic (TOPRO3 negative and Annexin V positive) component (Figure 1E). Human cells displayed a slower kinetics, with necrosis peaking at 48–72 hours, minor apoptotic component, and, again, with the tumor cells being more sensitive than non-transformed, immortalized cells (Figure 1F).

A time-course of PARP and Caspase 3 cleavage confirmed that a minor apoptotic component appears only at late time points, after massive necrotic death has already taken place (Figure 1G). Accordingly, pre-treatment with the apoptosis inhibitor Z-VAD-FMK did not prevent Obatoclax-induced cell death (Figure 1H).

Previously published studies using different cell lines have suggested that Obatoclax may induce necroptosis, a regulated form of necrosis that depends on RIP1 kinase activity [25, 26]. However, a RIP1 inhibitor, necrostatin-1, was completely unable to prevent Obatoclax-induced necrosis in thyroid cancer cells (Figure 1H).

These data strongly suggest that, upon Obatoclax treatment, thyroid cancer cells undergo primarily classical necrosis.

### Obatoclax induces a block in autophagy, unrelated to cell death

It has recently been proposed that Obatoclax induces autophagy in a number of experimental systems, and that autophagy is integral to Obatoclax' cytotoxic activity [26–29].

**Figure 2:**
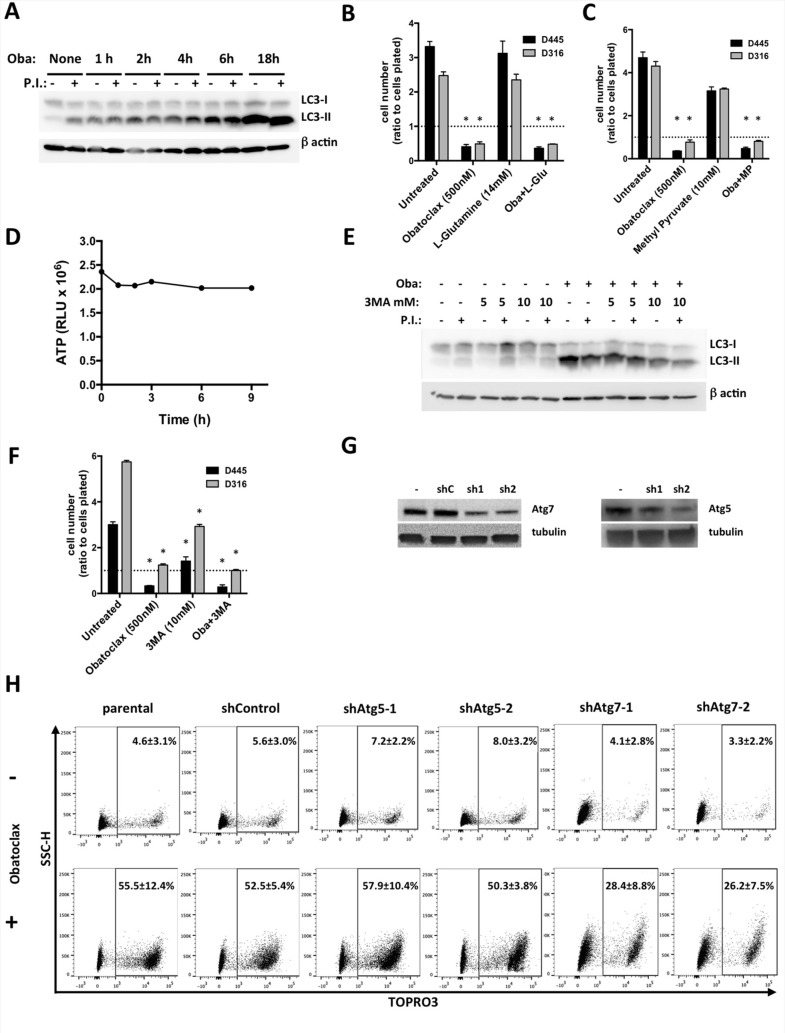
Obatoclax blocks late authophagy, unrelated to cell death (**A**) Western blot analysis of LC3 in D445 cells treated with Obatoclax (500 nM) for the specified times. Where indicated, a combination of lysosomal protease inhibitors (20 mM NH4Cl and 200 μM leupeptin, P.I.) was added 2h before collecting the cells (see Methods). (**B**, **C**) Relative cell counts of mouse thyroid cancer cells pre-treated for 1 hr with (B) L-Glutamine or (C) Methyl Pyruvate and then treated with Obatoclax for 24 hrs. (**D**) ATP levels in D445 cells treated with Obatoclax (500 nM) for the specified times. (**E**) Western blot analysis of LC3 in D445 cells treated with Obatoclax in the presence or absence of 3MA. (**F**) Relative cell counts of mouse thyroid cancer cell lines treated with Obatoclax in the presence or absence of 3MA. (**G**) Western blot analysis of D445 cell clones silenced for *Atg7* and *Atg5*. (**H**) Flow cytometric analysis of necrosis in D445 cells (parental, empty lentiviral vector, and silenced clones) treated with Obatoclax (500 nM) for 24 hrs. Numbers superimposed on the flow cytometry panels represent mean ± sd of three or more replicate experiments. Bars in graphs correspond to standard deviation.

Indeed, our time course analysis of LC3 lipidation upon treatment of D445 cells with Obatoclax showed a strong increase in steady-state levels of LC3-II, indicating higher abundance of autophagosomes upon Obatoclax treatment (Figure 2A). An increase in autophagosome content could be due to induction of autophagy (higher formation and clearance of autophagosomes) or reduced autophagy rates (equal autophagosome formation but failure to degrade them). To discriminate between these two possibilities, we directly measured the autophagic flux, detected as the increase in LC3-II levels upon blockage of lysosomal degradation. In clear contrast with untreated cells where treatment with inhibitors of lysosomal proteolysis lead to marked increase in LC3-II levels (indicative of degradation of autophagosomes in lysosomes), we found that shortly after treatment with Obatoclax inhibition of lysosomal proteolysis did no longer increase LC3-II over the levels detected in these conditions in absence of inhibitors. These results confirmed that treatment with Obatoclax decreased autophagic flux by almost completely blunting degradation of autophagosomes by lysososomes and causing in this way autophagosome accumulation (increase in steady-state levels of LC3-II) (Figure 2A).

In principle, a block in autophagy completion could be detrimental (and lethal) to tumor cells through a variety of mechanisms, including a reduction in availability of recycled substrates for energy production, or an unsustainable accumulation of vesicles in the cytoplasm, creating a *de facto* “traffic jam”.

We tested the ability of two readily available energy substrates, L-Glutamine and Methyl Pyruvate [30], to rescue the hypothetical energy deficit induced by Obatoclax in thyroid cancer cells, and found that neither compound could prevent or reduce the extent of necrotic death (Figure 2B, 2C). Furthermore, we did not detect any appreciable changes in available ATP in cells treated with Obatoclax over the course of several hours, even at time points already showing massive cell death (Figure 2D).

These data clearly indicate that the block in autophagy does not cause an energy crisis leading to necrosis.

If necrosis is a consequence of the excessive accumulation of autophagic vesicles, then an inhibitor of autophagosome formation should at least partially reduce cell death. We first established that 10 mM 3-methyladenine (3MA), an inhibitor of class III PI3K [31], was sufficient to significantly reduce the levels of LC3-II accumulated upon Obatoclax treatment, confirming that this concentration of 3MA was sufficient to reduce autophagosome production (Figure 2E). However, when cells were pre-treated with 3MA, Obatoclax was still able to kill them with unaltered efficacy (Figure 2F). Interestingly, also 3MA alone was able to significantly reduce cell growth, suggesting that thyroid cancer cells need a basal level of autophagy for survival and proliferation.

Finally, we used shRNAs targeting two key autophagy players, Atg5 and Atg7, to genetically block autophagy. While Atg5 downregulation did not protect thyroid cancer cells from the lethal effects of Obatoclax treatment, shAtg7 reduced the number of dying cells by approximately 50% (Figure 2G, 2H).

Taken together, these data show that the inhibitory effects of Obatoclax on the late steps of autophagy are independent of those on cell survival, and suggest that Atg7 might have autophagy-independent functions that are necessary for the ability of Obatoclax to kill thyroid cancer cells.

The notion that Obatoclax blocks late autophagy steps prompted us to test whether its effect might be amplified by nutrient starvation, which increases dependence on autophagy. As predicted, we found that starved cells are significantly more sensitive to Obatoclax than cells grown in complete medium (Supplementary Figure S2).

### Obatoclax localizes to lysosomes

**Figure 3:**
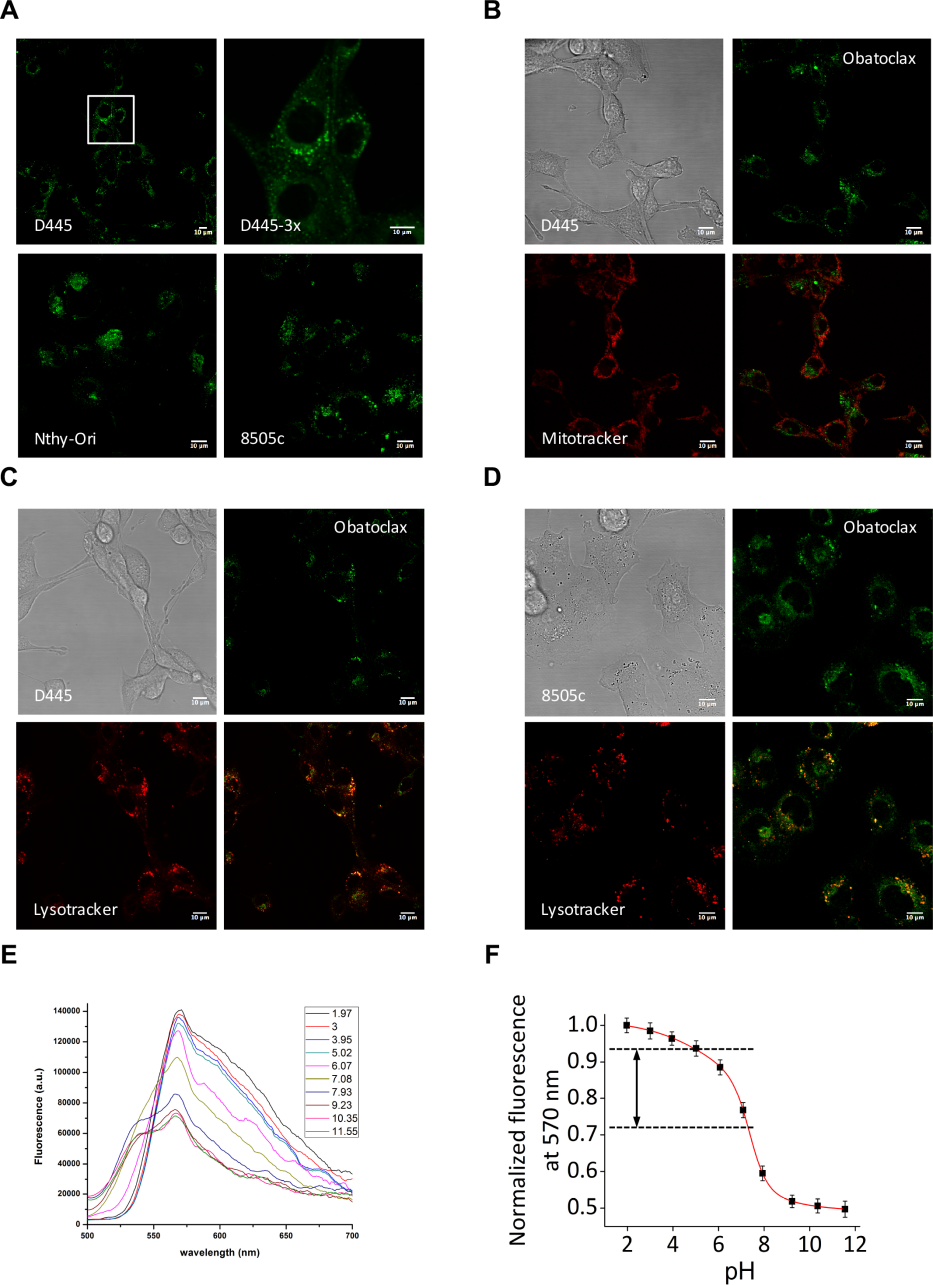
Obatoclax autofluorescence reveals its accumulation in lysosomes (**A**) Obatoclax autofluorescence visualized in the green channel as cytoplasmic puncta in mouse and human thyroid cells. (**B**) Obatoclax puncta do not co-localize with the mitochondria. (**C**, **D**) Obatoclax co-localizes with the lysosomes in (C) mouse and (D) human thyroid cancer cells. Bars: 10 μm. (**E**) Fluorescence emission spectra of Obatoclax measured at different pH values. (**F**) Dependence of the fluorescence intensity of Obatoclax on pH. Fluorescence signal at different pH values was normalized at 570 nm. Bars in graphs correspond to standard deviation.

We exploited Obatoclax autofluorescence to determine its subcellular localization in thyroid cells. Confocal imaging of live cells within a few minutes of treatment showed a cytoplasmic punctate pattern in both mouse and human cell lines (Figure 3A). These puncta were readily detected in both the FITC and the PI channels, but they did not survive fixation, thus hindering our ability to perform colocalization studies by immunofluorescence. Based on the notion that Obatoclax was designed as a pan-BCL2 family inhibitor, we hypothesized that those puncta might correspond to mitochondria. However, confocal microscopy in live cells revealed no signal colocalization with Mitotracker (Figure 3B). Surprisingly, instead, Obatoclax was found to colocalize with lysosomes in both mouse (Figure 3C) and human (Figure 3D) thyroid cancer cells.

Given the acidic environment of lysosomes, we wondered whether Obatoclax was only fluorescent at low pH conditions, and, as a consequence, whether we might just be unable to detect its presence in other cellular compartments due to a loss of fluorescence. Thus, we measured Obatoclax' fluorescence emission spectrum at different pH values and found that fluorescence of Obatoclax is indeed dependent on pH (Figure 3E). The fluorescence intensity changed 2-fold with the pH changes in the range of 2–12 (Figure 3F). Highest fluorescence was observed in acidic environment. However, while acidic conditions increased Obatoclax fluorescence emission, the difference between fluorescence intensity at cytoplasmic and lysosomal pH values was less than 25% (Figure 3F), suggesting that, in fact, Obatoclax was rapidly and exclusively trapped in lysosomes.

### Obatoclax affects lysosome structure and properties

**Figure 4:**
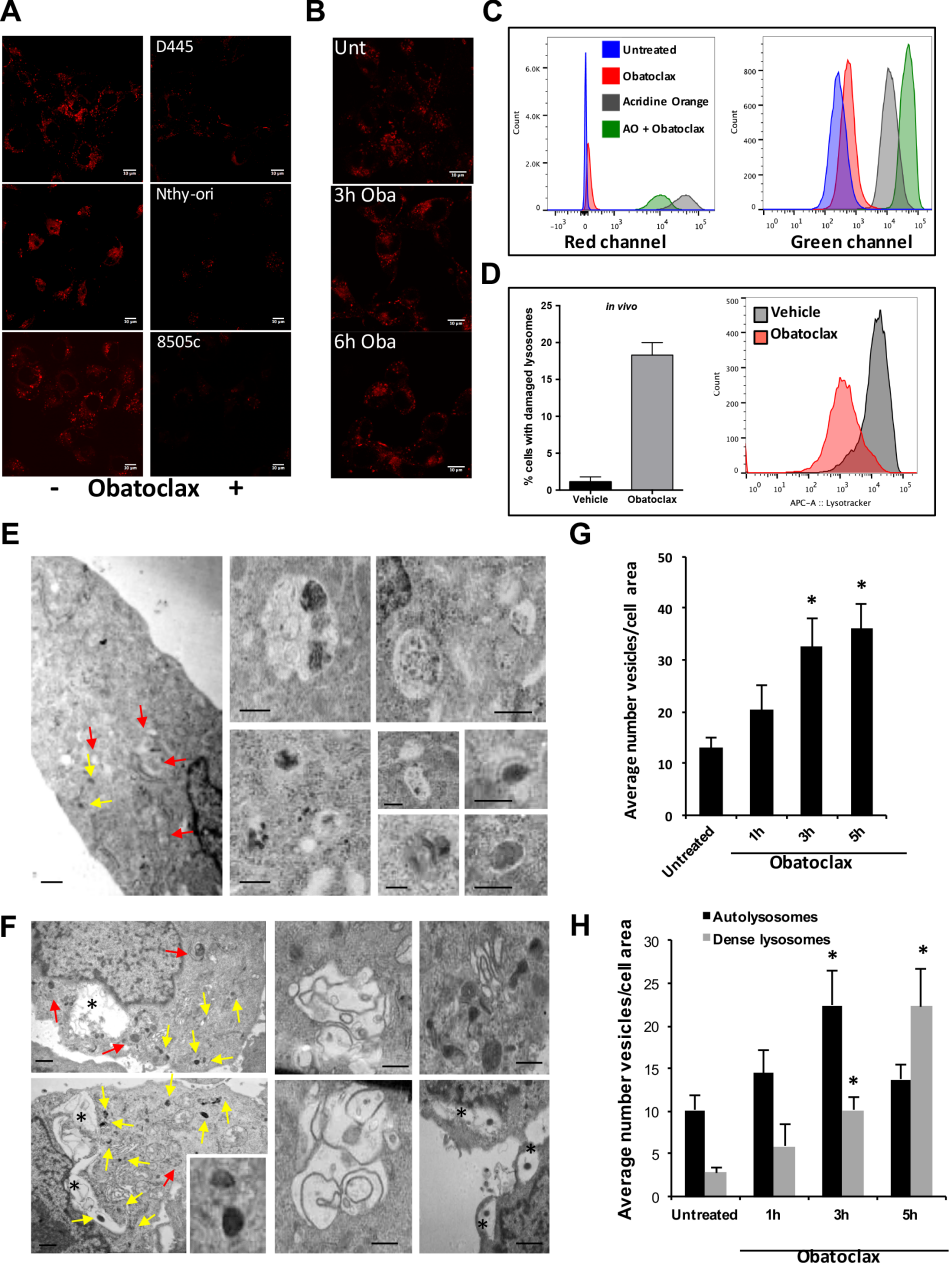
Obatoclax induces morphological and functional alterations in lysosomes (**A**) Lysotracker signal is lost after 30 minutes of Obatoclax treatment (500 nM for mouse cells, 1 μM for human cells) of thyroid cancer cells. (**B**) Immunofluorescence staining for LAMP2 in D445 cells treated with Obatoclax (500 nM) for the specified times. Bars: 10 μm. (**C**) Flow cytometric analysis of D445 cells pre-loaded with Acridine Orange (AO) and treated with Obatoclax (500 nM). Emission fluorescence was detected in the PerCP (Red) and FITC (Green) channels. (**D**) Flow cytometric analysis of Lysotracker fluorescence intensity in thyroid cells dissociated from tumors developed by [*Pten*,*Trp53*]^thyr−/−^ mice. Mice were treated with Obatoclax (5 mg/kg) for 6 days and loaded with Lysotracker before analysis. (**E**, **F**) Ultrastructural analysis of D445 cells untreated (E) or treated for 5 hrs (F) with 500 nM Obatoclax. Representative electron microscopy of cell sections; insets on the right show higher magnification examples of the different types of autolysosomes (red arrows) and dense lysosomes (yellow arrows). Bars: 2 μm (cytosolic regions) or 0.2 μm (insets). Asterisks (*) denotes large vacuolated regions often containing dense lysosomal-like structures. (**G**, **H**) Morphometric analysis of TEM images to calculate the average number of vesicles per cell area (G), and the number of autolysosomes (single membrane vesicles of light density with cargo inside) or dense lysosomes (high density smaller single membrane vesicles). Values are *n* = 6–9 individual cells and > 50 vesicles counted. Differences with untreated samples are significant for *p < 0.05. Bars in graphs correspond to standard deviation.

We noticed that if Obatoclax-treated cells were analyzed 30 minutes or more after drug exposure, the Lysotracker signal was lost in both mouse and human thyroid cancer cells (Figure 4A). To better characterize this phenomenon, we performed time-lapse imaging and found that while Lysotracker-positive puncta were stable for at least 40 minutes when imaging untreated cells, the Lysotracker signal rapidly disappeared in cells exposed to Obatoclax, suggesting that this compound alters lysosomal membrane properties, leading to loss of these organelles' ability to trap Lysotracker (Supplementary Movies S1–S4).

To assess the extent of damage to lysosomes and determine whether the loss of Lysotracker retention was the result of complete loss of lysosomes, we performed immunofluorescence experiments to detect LAMP2, an abundant lysosomal membrane component. We found that LAMP2 punctate pattern was retained for at least 6 hours after Obatoclax treatment, suggesting that this compound does not cause complete lysosomal destruction (Figure 4B).

To complement the experiments described above, we used Acridine Orange (AO) to examine lysosome stability. AO is a cationic, lipophilic fluorochrome capable of accumulating in lysosomes due to the acidity of these organelles. When AO is in its free monomeric form, it emits a green signal if excited by a blue laser. However, when the dye becomes concentrated in the lysosomes, it oligomerizes and the fluorescent signal emitted exhibits a shift towards the red. D445 cells treated with Obatoclax in the absence of AO display a slight shift of the fluorescence peak to the right in both the red and the green channels, as a consequence of Obatoclax autofluorescence (Figure 4C). When we preloaded cells with AO, treated them with vehicle or Obatoclax, and finally analyzed them by flow cytometry, we observed, in cells treated with Obatoclax, a marked shift of the red fluorescence peak to the left compared to cells treated with vehicle. At the same time, we observed a fluorescence peak shift to the right in the green channel. Taken together, these data indicate a loss of AO accumulation in the lysosomes and its redistribution within the cells (Figure 4C).

To establish whether Obatoclax-induced lysosomal alterations are also observed *in vivo*, we treated thyroid cancer-carrying [*Pten*,*Trp53*]^thyr−/−^ mice with vehicle or Obatoclax for 6 days, after which thyroid single cell suspensions were loaded with Lysotracker and analyzed by flow cytometry. Live thyrocytes in Obatoclax-treated mice exhibited a dramatic reduction in Lysotracker staining, strongly suggesting extensive structural and/or functional damage (Figure 4D).

Finally, we analyzed D445 cells by electron microscopy at different times after exposure to Obatoclax. Ultrastructural analysis revealed that Obatoclax causes a significant increase in the number of autophagy-related vesicular compartments, mostly as a result of higher number of secondary condensed lysosomes (Figure 4E–4H). Obatoclax treatment did not lead to an accumulation of immature autophagosomes (pre-lysosomal fusion), which indeed were rarely observed even in untreated cells, supporting an efficient autophagic flux in these cells. In untreated cells, the most abundant autophagy figures were autolysosomes (single membrane vesicles with cytosolic cargo at different stages of degradation). The total number of vesicles increased gradually during the first 5h after treatment (Figure 4G) along with a significant increase in the number of secondary dense lysosomes (Figure 4H). In addition, after 5h of treatment, very large vacuolar structures, often located in the perinuclear region, become visible (Figure 4F). Interestingly, secondary dense lysosomes were often located inside these large clear vesicles (note the dense round lysosomes inside the clear vesicles, denoted with an asterisk in Figure 4F), in possible support of activation of lysophagy upon lysosome disruption.

These results strongly suggest that Obatoclax causes a late block in lysosomal function by rendering lysosomes unable to fully digest their content. It is also tempting to hypothesize that the large vesicles engulf damaged lysosomes in an attempt to isolate them from the intracellular environment.

### Lysosome acidity is required for necrotic response

**Figure 5:**
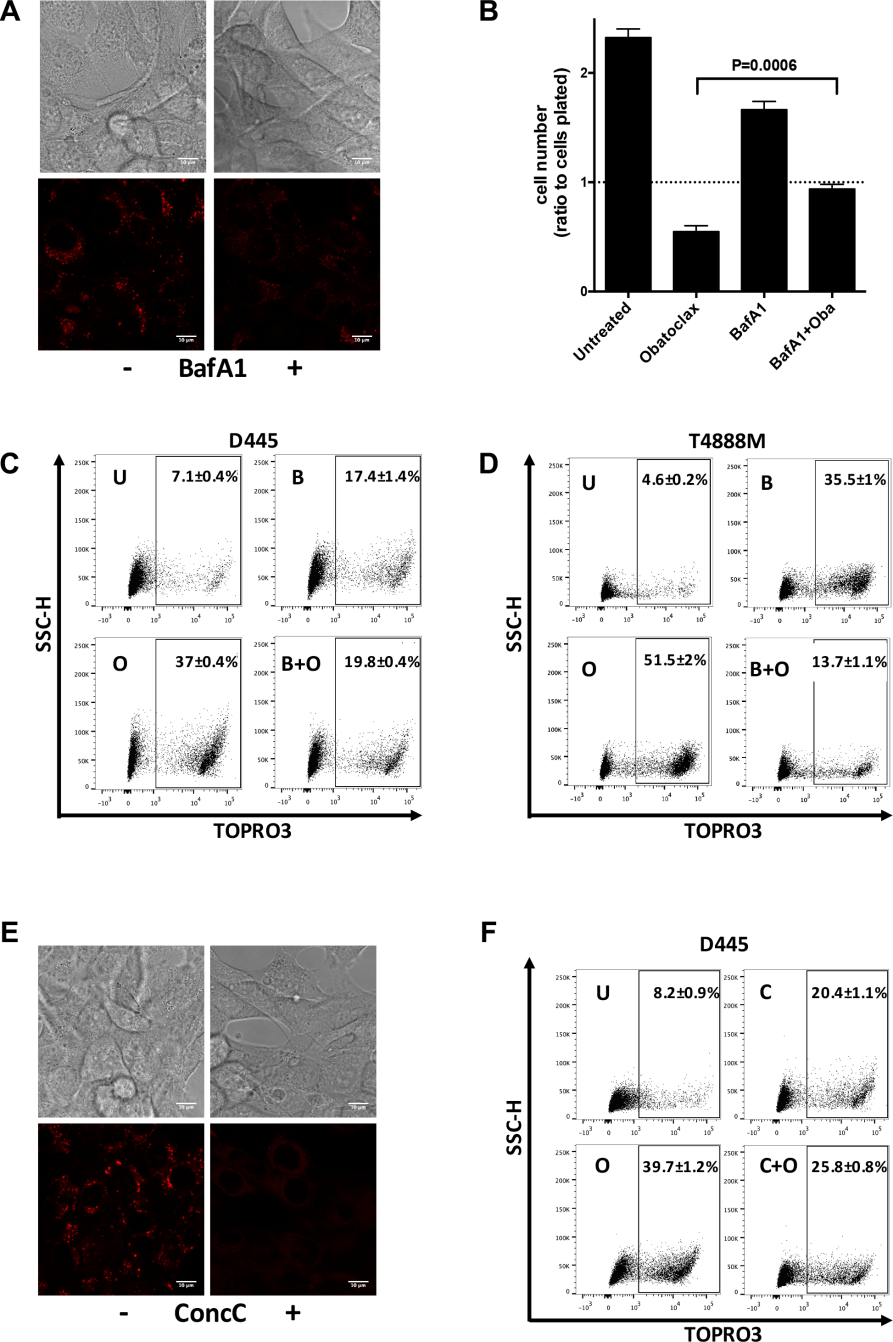
Lysosomal acidic environment is critical for necrotic death induced by Obatoclax (**A**) Lysotracker detection in D445 cells cultured with or without Bafilomycin (BafA1, 10 nM) pre-treatment (4 hrs). (**B**) Relative cell number of D445 cells pre-treated with BafA1 (10 nM) for 2 hrs and then exposed to Obatoclax (500 nM) for 16 hrs, in the presence of BafA1. (**C**, **D**) Flow cytometric analysis of necrosis in mouse thyroid cancer cells pre-treated with BafA1 (10 nM) for 4 hrs and exposed to Obatoclax (500 nM) for 10 hrs. (**E**) Lysotracker detection in D445 cells cultured in the presence or absence of Concanamycin C (ConcC, 25 nM) for 4 hrs. (**F**) Flow cytometric analysis of necrosis in D445 cells pre-treated with Concanamycin C (25 nM) for 4 hrs and exposed to Obatoclax (500 nM) for 10 hrs. Bars: 10 μm. Bars in graphs correspond to standard deviation. Numbers superimposed on the flow cytometry panels represent mean ± sd of three or more replicate experiments.

Lysosome membrane permeabilization (LMP) causes cell death through the acidification of the cytosol and/or the release of active cathepsins in the cytoplasm. Therefore, we sought to determine whether lysosomal acidity is important for the cells' necrotic response to Obatoclax. We first tested the ability of Bafilomycin A1 (BafA1), a V-ATPase inhibitor, to prevent the acidification of lysosomes. D445 cells were incubated with Lysotracker in the presence or absence of BafA1 and analyzed by microscopy. BafA1 was indeed able to prevent the acidification of lysosomes, as determined by the loss of Lysotracker signal (Figure 5A).

Thus, we pre-treated D445 cells with BafA1 for 2 hours before exposing them to Obatoclax and assessing their cell number after 16 hours. Lysosome neutralization significantly reduced cell proliferation in cells not treated with Obatoclax, suggesting that full lysosomal function is required for proper cell growth. Strikingly, Obatoclax ability to kill thyroid cancer cells was severely reduced in the presence of BafA1 (Figure 5B).

D445 and T4888M cells were also treated and collected for cell death analysis by flow cytometry. While BafA1 alone caused an increase in the percentage of dead cells, Baf-1 was also able to drastically reduce the extent of cell death in samples co-treated with Obatoclax (Figure 5C, 5D). In order to confirm that this rescue was in fact caused by the neutralization of lysosomes prior to Obatoclax treatment, we replicated these experiments using another V-ATPase inhibitor, Concanamycin C (ConcC). Again, we first confirmed that ConcC was able to prevent lysosomal acidification as detected by Lysotracker loss in D445 cells (Figure 5E). When cells were analyzed by flow cytometry, we found that ConcC alone, like BafA1, caused an increase in the percentage of cell death in comparison to untreated cells and that, again, pre-treatment with ConcC partially rescued the killing effect of Obatoclax (Figure 5F). Therefore, it appears that an intact lysosomal acidic environment is critical for the induction of cell death by Obatoclax.

### Obatoclax does not cause massive lysosomal permeabilization

**Figure 6:**
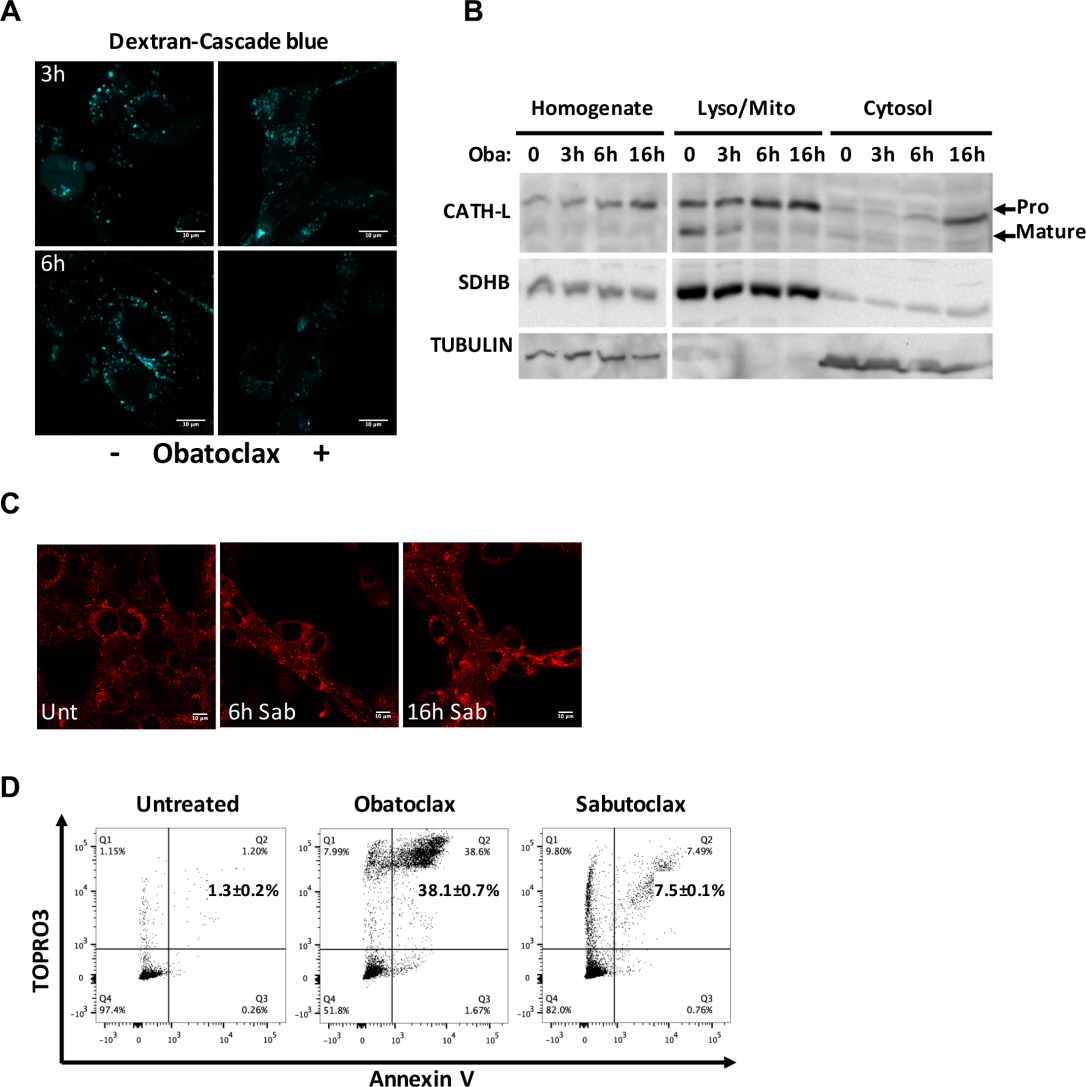
Obatoclax does not induce massive LMP, and its effect is independent of BCL2 proteins (**A**) Fluorescent dextran visualized in D445 cells treated with Obatoclax for the specified times. Cells were pulsed with dextran-cascade blue for 4 hours before being washed and chased in culture medium for 1 hour. After the chase, cells were incubated with Obatoclax. (**B**) Western blotting analysis of Cathepsin L maturation and localization in cell fractions from D445 cells treated with Obatoclax for the specified times. Lyso/Mito: Lysosome/mitochondria rich; Oba: Obatoclax. SDHB and Tubulin are markers of mitochondria and cytosol, respectively. (**C**) Lysotracker detection in D445 cells treated with Sabutoclax (Sab, 1 μM) for the specified times. (**D**) Flow cytometric analysis of cell death in D445 cells treated with Obatoclax and Sabutoclax (500 nM each) for 24 hrs. Numbers superimposed on the flow cytometry panels represent mean ± sd of three or more replicate experiments. Bars: 10 μm.

To understand whether the acidifying effect of proton leakage is accompanied by massive lysosomal membrane permeabilization, D445 cells were loaded with Cascade blue-labeled dextran (10 kDa), treated with vehicle or Obatoclax, and analyzed by microscopy after 3 and 6 hours (Figure 6A). The untreated samples showed a distinct punctate pattern, due to the endosomal and lysosomal loading of dextran after entering the cells through endocytosis. In cells treated with Obatoclax, no change in dextran localization was observed at 3 hours post-treatment, while a moderate loss of signal was evident at 6 hours, when significant necrosis is already underway (Figure 1E).

As a parallel approach to assess LMP, we performed cell fractionation studies to detect the possible release of lysosomal cathepsins into the cytosol, indicating extensive damage of the lysosomal membrane.

Cells were treated with Obatoclax and lysed at different time points to collect whole cell homogenates, a fraction enriched for lysosomes and mitochondria, and a clean cytosol fraction. When we used Western blotting to detect the relative levels of cathepsin L in these fractions, we found (i) no significant release of the precursor form into the cytosol until 16 hrs after treatment, and (ii) a strong decrease in the mature form of cathepsin L in the mitochondria/lysosome-enriched fractions upon treatment with Obatoclax, without any corresponding increase in the cytosol (Figure 6B). Given that the lysosomal acidic environment is essential for the proper maturation of cathepsins, the accumulation of pro-cathepsin L in the mitochondria/lysosome-enriched fraction upon Obatoclax treatment strongly suggests that the early neutralization of the organelle prevents cathepsin L processing, thus leading to the progressive reduction in abundance of the mature form.

Taken together, these results suggest that the primary, immediate effect of Obatoclax on the lysosomes is their neutralization, and that complete LMP occurs much later, thus excluding a major role for cathepsin leakage in inducing cell death after Obatoclax treatment.

### Determinants of obatoclax localization and activity

Taking advantage of the possibility of identifying neutralized lysosomes using fluorescent dextran, we tested whether an acidic environment is a prerequisite for the ability of Obatoclax to localize to the lysosomes. In fact, Obatoclax is a weak base, and thus, after entering into the lysosomes by diffusion, it could become protonated and trapped. Surprisingly, we found that pre-neutralization of the lysosomes with BafA1 did not prevent colocalization of dextran and Obatoclax, suggesting that Obatoclax accumulates into the lysosomes through a different, still unknown mechanism (Supplementary Figure S3).

To clarify whether Obatoclax ability to induce necrosis in thyroid cancer cells is associated with its design as a pan-BCL2 family inhibitor, we tested another, chemically unrelated, small molecule with the same target specificity, Sabutoclax.

Strikingly, Sabutoclax did not induce lysosomal destabilization as measured by the extent of Lysotracker staining (Figure 6C), and was unable to induce massive necrosis of thyroid cancer cells (Figure 6D).

These data strongly suggest that Obatoclax cytotoxic activity is not associated with inhibition of the BCL2 family members.

### Lysosomal damage as a therapeutic strategy for thyroid cancer

If Obatoclax induces thyroid cancer cells necrosis through its ability to neutralize and permeabilize lysosomes, then other well-established lysosomotropic agents should be able to produce similar effects.

We first tested the ability of Mefloquine to mimic Obatoclax activity. Mefloquine is an anti-malarial compound that has been shown to localize to and disrupt lysosomes in acute myeloid leukemia cells [32].

**Figure 7:**
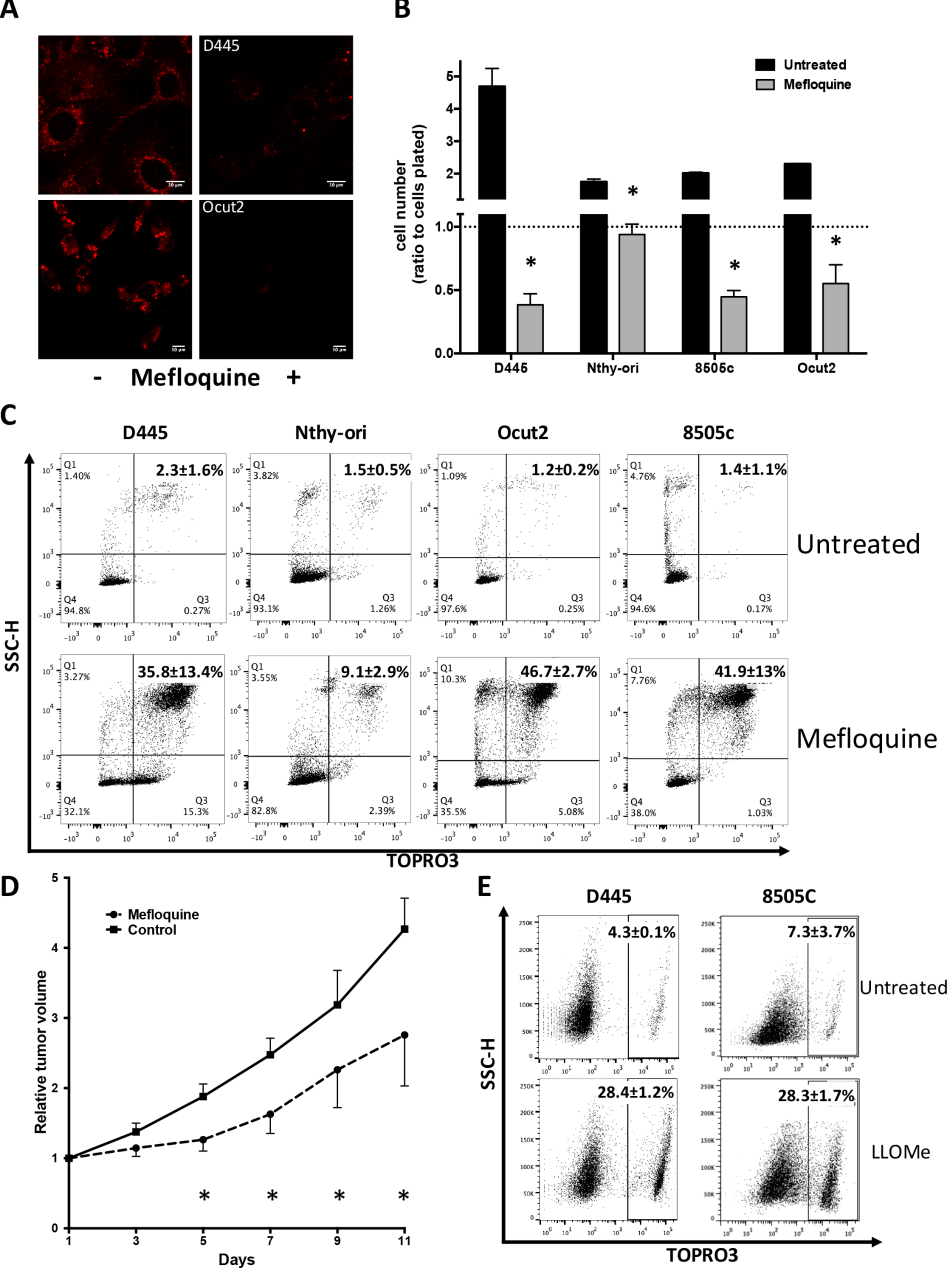
Mouse and human thyroid cancer cells are sensitive to lysosomal destabilization (**A**) Lysotracker detection in mouse and human thyroid cancer cells treated with Mefloquine (20 μM) for 60 min. (**B**) Relative cell counts and (**C**) flow cytometric analysis of necrosis in mouse and human immortalized (Nthy-ori) and transformed thyroid cells treated with Mefloquine (20 μM) for 24 hr. Bars in graph correspond to standard deviation. (**D**) Relative tumor volume (± SEM) of D445 allografts treated with Mefloquine (75 mg/kg) for 13 days. Asterisks mark time points where *P* < 0.03. (**E**) Flow cytometric analysis of necrosis in mouse and human thyroid cancer cells treated with LLOMe (2 mM) for 24 hrs. Numbers superimposed on the flow cytometry panels represent mean ± sd of three or more replicate experiments.

Like Obatoclax, Mefloquine was able to cause rapid loss of Lysotracker signal in both mouse and human thyroid cancer cells (Figure 7A) and to significantly reduce the number of treated cells below the plating baseline after 24 hours, indicating actual killing activity. In contrast, Mefloquine displayed mostly a cytostatic effect on normal, immortalized thyroid cells (Figure 7B).

These data were further confirmed by flow cytometric analysis showing massive cell death in mouse and human thyroid cancer cells, while this effect was significantly lower in immortalized human thyroid cells (Figure 7C).

We next tested the ability of Mefloquine to target thyroid tumors grown as allografts in immunocompetent mice. D445 cells were injected in the flank of syngeneic mice, which were randomized into a control and a treated cohort when tumors reached 200 mm^3^. Tumors in Mefloquine-treated mice grew with a significantly slower kinetics compared to vehicle-treated controls (Figure 7D).

Finally, we tested the effect of another lysosome-destabilizing compound, the dipeptide methyl ester Leu-Leu-OMe (LLOMe) [33], on the viability of mouse and human thyroid cancer cells. Once again, we found that thyroid cancer cells are extremely sensitive to lysosome-targeting agents, and respond to treatment with massive cell death (Figure 7E).

These data demonstrate that the sensitivity of thyroid cancer cells to Obatoclax is just one aspect of a more general sensitivity to lysosome-destabilizing agents, which are also effective *in vivo*, in immunocompetent, relevant preclinical models.

## DISCUSSION

Obatoclax is a pan-inhibitor of the anti-apoptotic members of the BCL-2 family of proteins, designed to prevent anti-apoptotic proteins from sequestering the apoptosis effectors BAX and BAK, and as such it should induce apoptosis. In fact, several groups have reported apoptotic response in cells treated with Obatoclax [24, 34–37]. However, a number of recent studies have suggested that Obatoclax induces, in addition to or instead of apoptosis, other cell death mechanisms, including toxic autophagy and necroptosis [25–29, 38].

Conversely, our data demonstrate that, at least in thyroid cancer cells, Obatoclax induces simple necrosis through the destabilization of lysosomes.

In reconciling these discrepancies, it is important to emphasize that the notion that Obatoclax generates auto-fluorescence was “buried” within the legend to a supplementary figure in the original article describing Obatoclax [24], and thus most reports have ignored this critical property. We have found that Obatoclax auto-fluorescence strongly interferes, in a dose-dependent manner, with the detection of both propidium iodide (PI) and FITC fluorescence, thus leading to misinterpretation of classical PI/Annexin V-FITC-based apoptosis assays, and of immunofluorescence detection of autophagic vesicles via FITC-linked antibodies. Furthermore, in most reports, the concept that Obatoclax induces autophagy derived from the detection of increased LC3-II levels after Obatoclax treatment. However, these experiments were performed without a critical control to assess autophagic flux (cells treated with or without lysosomal protease inhibitors), which would have shown (as our data do) that the LC3-II increase is caused by a block of autophagosome lysosome clearance (decreased autophagic flux) and not by enhanced autophagic flux.

Although the wide-range auto-fluorescence properties of Obatoclax have hampered our ability to perform additional critical experiments (for example to measure cytoplasmic pH changes upon treatment), we have been able to use dyes with fluorescence properties not impacted by Obatoclax to analyze cell death properties, and to harness Obatoclax auto-fluorescence to follow its distribution within the cell. Using these approaches, we have clearly shown that, in thyroid cancer cells, Obatoclax accumulates in lysosomes and causes necrosis as a result of massive lysosomal dysfunction.

Two aspects of these data are particularly relevant: first, the lower sensitivity of non-transformed thyroid cells to Obatoclax killing activity, which predicts a possible therapeutic window in the clinical setting, and an overall higher sensitivity of cells carrying mutations that activate MAPK signaling, compared to those driven by increased PI3K signaling. The molecular bases for this driver selectivity remain to be addressed.

From a mechanistic standpoint, the concurrent accumulation of autophagic vesicles consequent to their impaired clearance does not appear to play any significant role in the induction of cell death, since neither pharmacological (3MA) or genetic (sh *Atg5*) autophagy inhibition, nor supplementation with readily available energy substrates impaired Obatoclax ability to kill thyroid cancer cells. Remarkably, we found that genetic inhibition of *Atg7* significantly reduced the extent of cell death upon Obatoclax treatment. This result is in line with recent studies showing that ATG7 deficiency suppresses, through autophagy-independent mechanisms, cell death induced by lysosomal photodamage, raising the intriguing hypothesis that Atg7 might regulate lysosomal membrane composition and permeability [39, 40].

Our data establish that Obatoclax concentrates into the lysosomes, leading to their neutralization. This process might be mechanistically explained by the notion that Obatoclax has also been shown, at least *in vitro*, to possess anion carrier activity and to be able to function as an antiport for Cl^−^ and HCO_3_^−^ in model liposomes [41]. This mechanism might thus lead to the efflux of Cl^−^ from lysosomes, thus reducing the activity of the V-ATPase by increasing voltage across the lysosomal membrane [42], and to an influx of HCO_3_^−^, causing the rapid increase of lysosomal pH we have observed in cells exposed to Obatoclax. Accordingly, the ability of V-ATPase inhibitors to induce rapid cell death on their own (although at lower levels than Obatoclax) underlines the critical role of proper lysosome pH for thyroid cancer cell viability.

However, our data also demonstrate that pre-existing lysosomal acidity, while not required for Obatoclax trapping, is essential for its killing effects, suggesting that proton leak into the cytoplasm might be the primary cause of the necrotic response. At the same time, LAMP2 immunolocalization, as well as fluorescent dextran chase and cellular fractionation experiments concur in suggesting that Obatoclax-loaded lysosomes do not become extensively ruptured, and that release of lysosomal cathepsins is not responsible for cell death induction as seen, instead, in other examples of lysosomal membrane permeabilization [43–47]. Thus, these data suggest the possibility that both functional lysosomal impairment due to their neutralization, and, perhaps, a sudden H+ release into the cytoplasm participate in the induction of the necrotic response after Obatoclax exposure.

There are two important questions to which we still cannot provide a conclusive answer: the mechanism of Obatoclax trapping into the lysosomes, and whether the lysosome-damaging activity requires its interaction with BCL-2 family members. Although the weak base properties of Obatoclax would support a mechanism of protonation-based trapping into the lysosomes, as observed for other lysosomotropic amines [48], we found that lysosomal neutralization did not affect Obatoclax localization, suggesting that a different mechanism is responsible for its sequestering.

The finding that Sabutoclax, another pan-BCL-2 family inhibitor, structurally different from Obatoclax, does not disrupt lysosomal function and does not induce cell death, argues against a role for these anti-apoptotic proteins in Obatoclax localization and cell-killing activity, although this possibility cannot be completely ruled out.

While this manuscript was being finalized for submission, three other groups have published reports that are relevant to our findings. Broecker-Preuss and co-workers have provided evidence that Obatoclax can effectively target thyroid cancer cells, inducing cell death with mixed apoptotic, necrotic, and autophagic features [49]. Our data, however, significantly extend these findings, providing a defined mechanistic framework for Obatoclax-induced lysosomal damage and cell necrosis. Stamelos and co-workers have recently found that Obatoclax accumulates into lysosomes of ovarian cancer cells, leading to their alkalization [50], providing an independent corroboration of our data. However, in contrast to our findings, apoptosis appeared to be the primary cause of cell death in several of the cell lines analyzed, and in general cell death occurred late, after 48 to 72 hours from Obatoclax treatment, while we show evidence of necrosis in thyroid cancer cells as early as six hours post-treatment. Furthermore, Stamelos found that BafA1 did not reduce Obatoclax-dependent cell death, which may be explained by the fact that the two compounds were added simultaneously and not, as in our case, sequentially.

Finally, Yu and co-workers have shown that Obatoclax localizes to lysosomes in esophageal cancer cells, leading to impaired cathepsin expression (the effect on cathepsins had previously been reported also by Schwartz-Roberts *et al.* [51]) and promotion of cell death [52].

Most importantly, the unexpected Obatoclax mechanism of action we have characterized uncovers a general Achilles' heel of advanced thyroid cancer cells: in fact, these tumors are extremely sensitive to lysosomal destabilization and permeabilization, as shown by both the extensive necrosis induced in cell culture by both Mefloquine and LLOMe, and by the significant reduction of tumor growth *in vivo*, in an immunocompetent allograft system treated with Mefloquine.

In conclusion, we have identified a clear vulnerability of thyroid cancer cells representative of tumors with a dismal prognosis. Our data thus warrant further in depth analysis of the mechanisms controlling lysosome fragility in advanced tumors and the clinical impact of their targeting with lysosomotropic agents.

## MATERIALS AND METHODS

### Cell lines

Mouse cell lines were established from tumors developed in *Kras* ^G12D^, *Trp53* ^thyr−/−^ mice [11] and [*Pten*, *Trp53*]^thyr−/−^ mice [10] as previously described. Mouse cell lines and human cell lines FTC-133 and 8505c were grown in DMEM (HyClone) supplemented with 10% FBS (HyClone) and Mycozap Plus-CL (Lonza). Cal62, Nhy-ori-3-1, and OCUT-2 cells were grown in RPMI (HyClone) supplemented with 10% FBS and Mycozap Plus-CL. All cells were maintained at 37°C with 5% CO_2_. Cell identity was validated by amplifying and sequencing genomic fragments encompassing their known mutations (see Table 1).

### Reagents

Reagents used for treatments included GSK1120212 (Selleck), Obatoclax mesylate (Selleck), ABT-263 (Selleck), Doxorubicin (Selleck), Z-VAD-FMK (R&D Systems), Necrostatin-1 (Tocris), L-glutamine (Gibco), Methyl-pyruvate (Sigma), 3-MA (Sigma), Concanamycin C (ThermoFisher) and Bafilomycin A1 (Sigma).

### Western blot and antibodies

Cells were homogenized on ice in RIPA buffer (Thermo Scientific) and supplemented with Halt Protease and Phosphatase Inhibitor cocktail (Thermo Scientific). Protein concentration was determined using the Pierce BCA protein Kit (Thermo Scientific). Primary antibodies included anti-cleaved PARP (Cell Signaling), anti-Caspase-3 (Cell Signaling), anti-ATG5 (Novus), anti-ATG7 (Cell Signaling), anti-LC3 (Cell Signaling), and anti-Cathepsin L (Santa Cruz). Signals were detected with HRP-conjugated secondary antibodies and chemiluminescence substrate (ECL, Millipore). Equivalent loading was confirmed with anti-β-actin (Sigma), anti- β-tubulin (Sigma), and anti-SHDB (Sigma) (for lysosome/mitochondria rich fractions).

### LC3 flux assays

Macroautophagy activity was estimated by immunoblot for LC3-II in cells untreated or treated with the lysosomal protease inhibitor mixture 20 mM NH4Cl/200 μM leupeptin for the last 2 h of the Obastoclax treatment. The level of LC3 lipidation was quantified as the ratio of the measured LC3-II to Actin levels. LC3 flux was quantified as the relative ratio of LC3-II/actin values between samples with or without the lysosomal proteolysis inhibitors [53, 54].

### Cell proliferation and cell death analysis

In order to assess cell number, cells were washed, harvested, and counted using a Z2 Coulter counter (Beckman Coulter). In rescue experiments, cells were pre-treated with Z-VAD-FMK, Necrostatin-1, L-glutamine, Methyl-pyruvate, and 3-MA for 1 hour prior to treatment with Obatoclax.

For apoptosis and necrosis analysis, cells were stained with Topro3 (Invitrogen). For specific experiments, staining with Annexin V-Alexa Fluor 350 (Invitrogen) for 15 min at room temperature was also performed before analysis. All experiments were done in duplicates. All samples were analyzed by flow cytometry within 1 hr of collection and staining using a Becton Dickinson LSRII System (BD Biosciences). Flow cytometry analysis was done on the FloJo platform.

### 3D spheroid assays

Cells were plated in 96-well round bottom, ultra-low attachment plates (Corning). Cells were allowed to grow for 4 days before being treated with 1 μM Obatoclax for 7 days. Cell viability was assessed by the acid phosphatase assay [55].

### ATP measurement

D445 cells were treated with Obatoclax for different time intervals and collected all at once. ATP measurements were done using an ATP Assay Kit (EnzyLight) according to manufacturer's instructions. Luminescence was read using a luminometer (Glomax 20/20 Luminometer, Promega) within 1 min after adding the reagent.

### shRNAs

D445 cells were transduced with shATG5 and shATG7 lentiviral particles (TRC Genome-Wide shRNA Collection developed by The RNA Consortium). Cells were selected for 72 hours with puromycin before plating and treating for flow cytometry analysis.

### Lysotracker studies

Lysotracker Deep Red (Life Technologies) was used to label lysosomes. Mouse cell lines were incubated with 50 nM Lysotracker for 30 min at 37°C, and human cell lines at a 100 nM concentration for 1 hr at 37°C. Medium was replaced before analysis, which was done with a Leica SP5 AOBS microscope.

For co-localization studies, cells were incubated with Lysotracker for 30 min before adding Obatoclax (500 nM). Images were taken in z-stacks smaller than 0.3 μm. within the first 10 minutes after adding the drug.

In order the assess the loss of Lysotracker signal upon Obatoclax treatment, D445 cells plated in a glass 8-chamber slide were incubated with Lysotracker (50 nM)) for 30 min. After replacing the medium, Obatoclax (500 nM) was added to the cells and imaging was started. Images were taken from untreated and treated chambers for every minute over a period of 40 minutes, and the Lysotracker fluorescence was detected in the Cy5 channel. These studies were done using an Inverted Olympus IX71 microscope.

### Mitotracker studies

MitoTracker Deep Red (Life Technologies) was used to label mitochondria in co-localization studies. Cells were incubated with MitoTracker (100 nM) for 30 minutes before medium was replaced. Cells were treated with Obatoclax (500 nM) and imaging was done within the first 10 minutes. A Leica SP5 AOBS microscope was used and images were taken in z-stacks smaller than 0.3 um.

### Immunofluorescence

After treatment with Obatoclax (500 nM), cells were washed in PBS and fixed in Methanol for 1 hour at −20°C. Fixed cells were washed in PBS and blocked in 1% BSA PBS-T with 2% goat serum for 1 hour at room temperature. After the blocking step fixed cells were incubated with anti-LAMP2 (Abcam) in 1% BSA PBST for 1 hour at room temperature. After incubation cells were washed and incubated with anti-rat Alexa Fluor-647 conjugate (Life Technologies) in 1% BSA PBST for 1 hour at room temperature. DAPI was added for nuclear stain. Images were taken using a Leica SP5 AOBS microscope.

### Dextran tracing

Cells were pulsed with 5 mg/ml of dextran-cascade blue 10,000 MW (Life Technologies) for 4 hours before being washed and chased in culture medium for 1 hour. After the chase, cells were incubated with Obatoclax (500 nM) for the specified times before being imaged using a Leica SP5 AOBS microscope.

### Acridine orange studies

Cells were incubated with Acridine Orange Hydrochloride (Sigma) at a 5 μg/ml final concentration for 30 minutes at 37°C, then washed with PBS and treated with Obatoclax (500 nM) for 1.5 hours before attached cells were collected by trypsinization for analysis. Untreated and treated cells were analyzed in the PerCP and FITC channels using a Becton Dickinson LSRII System (BD Biosciences).

### Cell fractionation and cytosol concentration

After treatment with Obatoclax (500 nM), cells were collected, resuspended in 25 mM sucrose and subjected to liquid nitrogen cavitation (35 psi) for 7 minutes and then homogenized. Homogenates were centrifuged for 2,500 g for 15 minutes to discard the postnuclear particulate (PNP) pellet. The resulting supernatant was centrifuged at 17,000 g for 10 minutes to collect the mitochondria/lysosome (M/L) rich fraction pellet. The supernatant was centrifuged at 100,000 g for 1 hour to discard the endoplasmic reticulum (ER) fraction pellet and obtain a pure cytosol. The cytosol fractions were concentrated using Amicon Centrifugal Filter 10K Devices (Millipore) following manufacturer's instructions. The M/L pellet was washed and resuspended in sucrose solution. All protein collections were supplemented with Halt Protease and Phosphatase Inhibitor cocktail (Thermo Scientific) and stored at −80°C until used for western blot analysis.

### Obatoclax fluorescence

Obatoclax fluorescence excitation and emission dependency on pH was measured as described in [56]. In brief, pH titrations of 4 μM Obatoclax solutions were performed using a series of Hydrion buffers with a range pH values from 1.97 to 11.55 (Micro Essential Laboratory). The excitation and emission spectra were measured using a FluoroMax-3 spectrofluorometer (Jobin Yvon). Fluorescence emission spectra were recorded from 485 to 780 nm (emission slit 4 nm) with excitation at 475 nm (excitation slit 2 nm).

### Transmission electron microscopy

Samples were fixed with 2.5% glutaraldehyde, in 0.1 M sodium cacodylate buffer. They were postfixed with 1% osmium tetroxide followed by 2% uranyl acetate, dehydrated through a graded series of ethanol, and embedded in LX112 resin (LADD Research Industries). Ultrathin sections were cut on a Reichert Ultracut E, stained with uranyl acetate followed by lead citrate and viewed on a JEOL 1200EX transmission electron microscope at 80 kv.

Autophagic vacuoles were identified using standard criteria [57, 58] and were catalogued as autophagolysosomes if they meet two or more of the following criteria: single membrane vesicles, with cytosolic material, of lighter density than surrounding cytosol, and absence of ribosomes associated to the membrane. Vesicles were considered “dense lysosomes” (secondary lysosomes) if they were single membrane, with density 3 times higher than the surrounding cytosol, and with amorphous/homogenous content. Morphometric analysis was performed in micrographs taken randomly by a technician uninformed of the nature of the samples. Cataloguing and quantification was done blindly in 6–9 cellular profiles and number of vesicles counted per condition ranged between 50–150 vesicles.

### *In vivo* studies

Mice harboring [*Pten, p53*]^thyr−/−^ tumors were treated with Obatoclax (5 mg/Kg) via i.p. injection once every day for 6 days. Tumors were dissected and digested with Liberase (Roche). Single cell suspensions were incubated with 100 nM Lysotracker Deep Red (Life Technologies) for 30 minutes before flow cytometry analysis.

8–10 week-old female wild type 129Sv mice were injected with 6 × 10^6^ D445 cells. When tumors reached a size between 200 and 250 mm^3^, mice were randomized to placebo and Mefloquine treatment groups (*n* = 20/group). Mefloquine was administered via oral gavage (75 mg/kg) once every day, and tumor volume was calculated from two-dimensional measurements using the following equation: tumor volume = (length × width^2^) × 0.5.

### Statistical analysis

Experiments were performed at least three times. Data were analyzed using the Prism software package. Differences with *P*-values < 0.05 were considered statistically significant.

## SUPPLEMENTARY FIGURES AND TABLES










