# Supplementary material for: Obatoclax kills anaplastic thyroid cancer cells by inducing lysosome neutralization and necrosis
Source: Oncotarget. 2016 Apr 30;7(23):34453–71. doi: 10.18632/oncotarget.9121 (PMC5085168; doi:10.18632/oncotarget.9121)
Supplement: Supplementary file 1 [file oncotarget-07-34453-s001.pdf]

Obatoclax kills anaplastic thyroid cancer cells by inducing lysosome neutralization and necrosis

Supplementary Materials

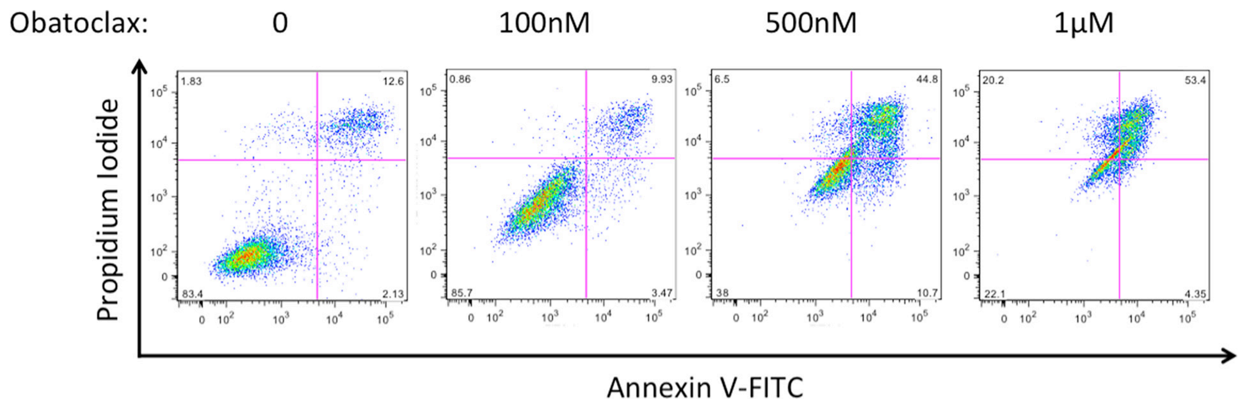

Supplementary Figure S1: Obatoclax autofluorescence affects apoptosis detection. Flow cytometric analysis of D316 cells treated with vehicle or different Obatoclax concentrations for 12 hours and then stained with Annexin V-FITC and Propidium iodide. Note the dose-dependent shift of the live cell population, which becomes fluorescent in both channels upon Obatoclax treatment, in a dose-dependent manner.

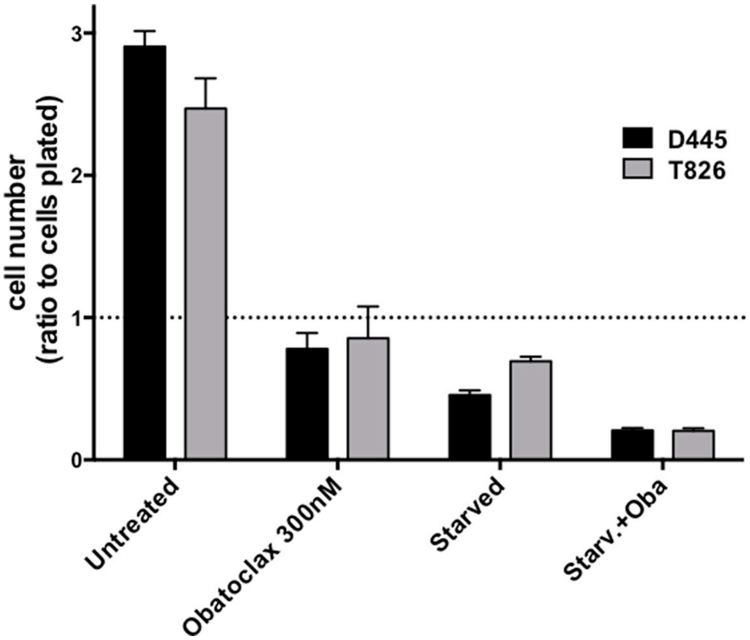

Supplementary Figure S2: Starved cells are more sensitive to Obatoclax. Relative cell numbers of mouse thyroid cancer cells treated with Obatoclax (300 nM) in complete medium or under starving conditions (HBSS containing 1% of normal medium) for 24 hrs.

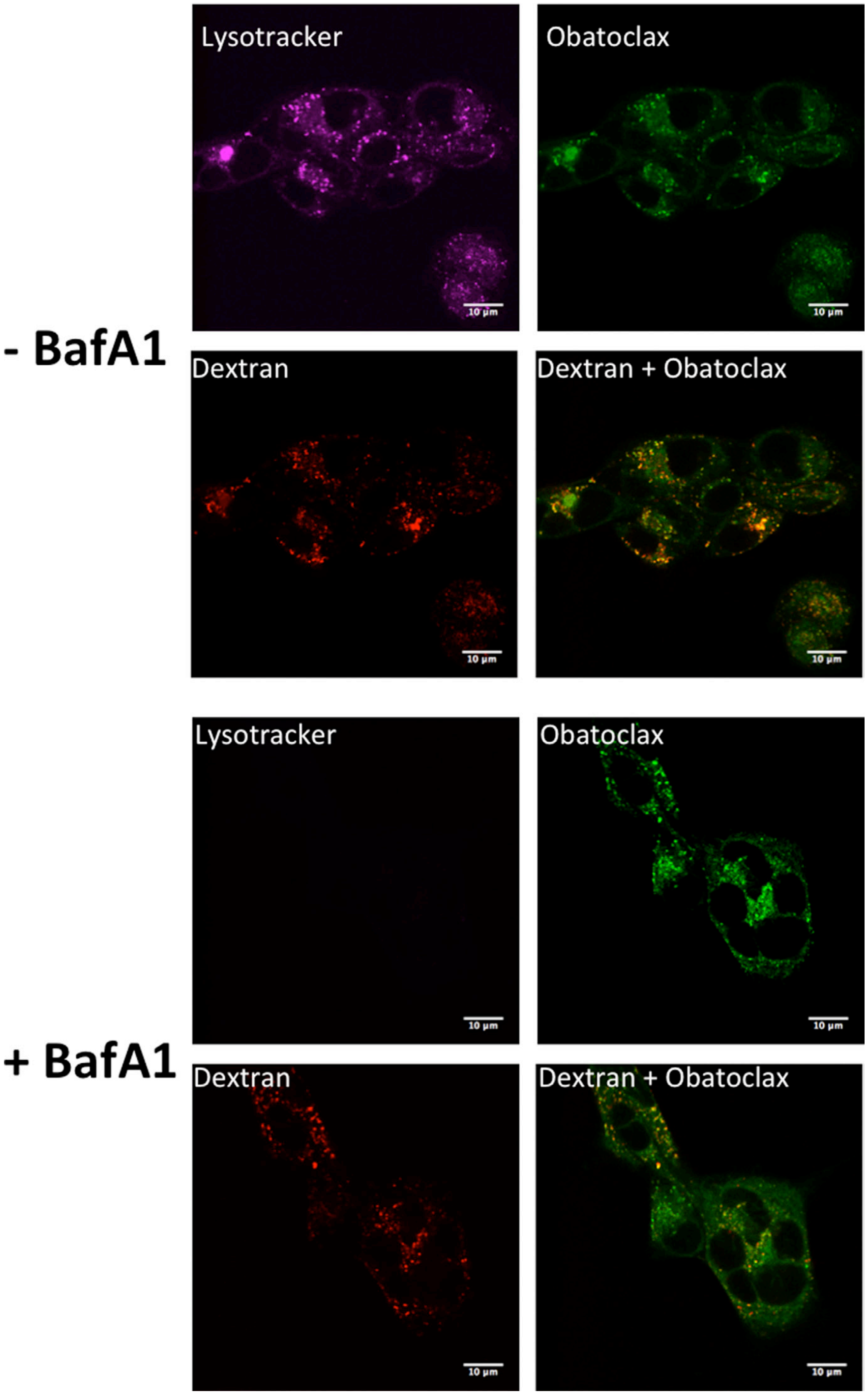

Supplementary Figure S3: Obatoclax localization to the lysosomes is independent of an acidic environment. Lysotracker, dextran and Obatoclax fluorescence visualized in D445 cells cultured in the absence or presence of Bafilomycin (BafA1) pre-treatment.

Supplementary Movie S1: Time-lapse microscopy (one image/min) of D445 cells loaded with Lysotracker, over the course of 40 min.

Supplementary Movie S2: Time-lapse microscopy (one image/min) of D445 cells treated with Obatoclax, over the course of 40 min.

Supplementary Movie S3: Time-lapse microscopy (one image/min) of D445 cells loaded with Lysotracker and then treated with Obatoclax, over the course of 40 min. Green channel: Obatoclax signal.

Supplementary Movie S4: Time-lapse microscopy (one image/min) of D445 cells loaded with Lysotracker and then treated with Obatoclax, over the course of 40 min. Red channel: Lysotracker signal.
